# Requirements for Universally Accessible Upper-Body Exercise Equipment: The Case of People with Spinal Cord Injuries in Korea

**DOI:** 10.1155/2023/6652703

**Published:** 2023-12-22

**Authors:** Kwang-Ok An, Myo-Jung Choi, Sung-Shin Kim, Bo-Ra Kang, Young-Hyeon Bae, Hyosun Kweon

**Affiliations:** ^1^Department of Healthcare and Public Health, Rehabilitation Research Institute, Korea National Rehabilitation Center, Republic of Korea; ^2^Department of Clinical Rehabilitation, Rehabilitation Research Institute, Korea National Rehabilitation Center, Republic of Korea

## Abstract

**Background:**

People with disabilities face considerable obstacles when exercising, which precludes them from the social and health benefits of physical activity. Especially for individuals with paraplegia with spinal cord injuries, it is necessary to maintain continuous participation in physical activity even after discharge, as it helps to maintain mobility and daily living activities through upper body strength. However, the participation rate of people with disabilities in physical activity in Korea is still low, mainly due to the lack of exercise equipment and facilities.

**Objectives:**

The aim of this study is to identify aspects that can be improved for better accessibility to exercise equipment for individuals with paraplegia with spinal cord injuries and to reach a consensus on possible guidelines for accessible exercise equipment.

**Methods:**

This study reviews and evaluated the usability of four existing upper-body exercise equipment for individuals with paraplegia with spinal cord injuries. To assess usability, task performance scores and time were measured, and a survey was conducted on safety and satisfaction. Based on these results, areas for improvement were identified. Through literature review, usability results, and opinions from various stakeholders, eight requirements for universal accessibility were proposed.

**Results:**

It is necessary to consider how wheelchair users access the exercise equipment. The access method to the exercise area (facility regulations, auxiliary equipment to be provided, etc.) and placement of exercise equipment should also be considered. Information such as explanations of the exercise equipment and how to use it should be located within the wheelchair user's field of vision. Considering the participation rate in sports for people with disabilities in Korea, it is necessary to explain the exact exercise equipment and exercise method. It is also necessary to consider how wheelchair users transfer from the wheelchair to the seat of the exercise equipment. Parts that require manipulation of each exercise equipment must be within the wheelchair user's range of motion. Various supports or assistive devices that provide body support according to each piece of equipment are needed. In addition to the wheelchair's own brake, it is necessary to provide a fixing device so that the wheelchair does not move during the exercise.

**Conclusion:**

For people with spinal cord injuries, the arm ergometer, aerobic exercise equipment, showed higher scores in performance, stability, and satisfaction compared to other exercise equipment. Among the strength exercise equipment, shoulder press had an effect on performance, seated lat pull-down had an effect on stability, and seated chest press had an effect on satisfaction. Therefore, when selecting exercise equipment, it is necessary to recommend aerobic and strength exercise equipment according to the preferences of people with spinal cord injuries. When developing strength exercise equipment, it is necessary to consider usability evaluation factors for individuals with spinal cord injury.

## 1. Introduction

In the past, exercises for people with disabilities were mostly medical-purpose activities (rehabilitation and physical therapy), but as their social participation increased, it expanded into activities for sustainable health management in the community [1, 2]. In general, exercise increases activity levels and reduces secondary health problems (early death, coronary heart disease, stroke, type 2 diabetes, colon cancer, or dementia) and health costs associated with disability. Exercise can also boost the self-confidence of people with disabilities by successfully allowing engagement in physical activities and providing social opportunities to interact with people without disabilities [3–9]. Especially for individuals with paraplegia with spinal cord injuries, it is necessary to maintain continuous participation in physical activity even after discharge, as it helps to maintain mobility and daily living activities through upper body strength [10]. However, the participation rate in exercise activities of people with disabilities in Korea is significantly lower than that of people without disabilities due to inaccessible facilities and exercise equipment and limited professional support [1].

In other countries, there are universal exercise equipment standards that ensure accessibility for both people with and without disabilities [11–13]. These standards can promote accessible exercise equipment, while providing a reference for manufacturers to design product lines considering accessible features for people with disabilities (high-contrast handles/lever/button, strap to add extra stability, adjustable seat, swing-away seat, or one-handed access for changing weight stacks) [14–16]. In Korea, research on these standards is still lacking. When people with disabilities exercise, they either partially modify the commercial exercise equipment developed for people without disabilities or use exercise equipment developed for people with disabilities. In these cases, either safety is not guaranteed, or the price of the exercise equipment is high.

Therefore, we aimed to improve the accessibility of exercise equipment for people with disabilities by establishing guidelines for the development of universal exercise equipment that can be used by both people with and without disabilities. This study focused on the accessibility of upper-body exercise equipment for people with spinal cord injuries. We conducted a standard review of exercise equipment and performed a usability evaluation of four types of existing upper-body exercise equipment. In addition, a multidisciplinary team composed of various stakeholders (persons with disabilities, clinicians, exercise equipment test analysis experts, exercise equipment developers, certification system managers, and people in charge of sports facilities) was established, and their opinions were collected to direct guideline development.

## 2. Materials and Methods

### 2.1. Formation for Multidisciplinary Team

A multidisciplinary team was formed with 28 stakeholders (persons with disabilities, clinicians, exercise equipment test experts, exercise equipment developers, certification system managers, and people in charge of sports facilities). The contents of the guideline development direction were conceived from the beginning of the study by synthesizing the results of previous research and literature research, and various opinions were collected through four discussions and brainstorming.

### 2.2. Review of Exercise Equipment Standards

To collect information related to the universal guidelines for exercise equipment, four standards were reviewed for all specifications regarding accessibility. Two domestic studies [17, 18] and two other studies [11, 19] were analyzed.

There are two types of safety criteria for exercise equipment in South Korea. The first is the safety criteria for stationary training equipment [17]. Stationary training equipment is used to promote the physical strength of users at home and in public places, such as sports associations, educational establishments, or hotels. Typical fitness equipment includes fixed-type training equipment, bench presses, treadmill, bicycle ergometer, stepper, rowing machines, and exercise sliders. “General safety requirements and test methods” consist of scope, references, terms and definitions, classification, safety requirements, test methods, care and maintenance, assembly instructions, test method, general instructions for use, and marking. The other safety criteria target outdoor exercise equipment [18]. It stipulates safety requirements, test methods, and label of outdoor exercise equipment that is freely accessible. It also includes safety requirements for materials used in manufacturing outdoor exercise equipment products and product structure and design but does not include safety requirements for installation and management after manufacturing. These two safety criteria include commercial fitness equipment, including the four types of exercise equipment selected for this study.

The European standard EN 957-1:2005 (stationary training equipment—part 1: general safety requirements and test methods) specifies the general safety requirements for stationary training equipment. It consists of scope, normative references, terms and definitions, classification, safety requirements, test methods, care and maintenance, assembly instructions, general instructions for use, and marking [19]. The EN 957-2:2003 specifies safety requirements for strength-training equipment in addition to the general safety requirements of EN 957-1. This part is applicable to stationary strength-training equipment with stack weight resistance or other means of resistance such as weight disks; elastic cords; hydraulic, pneumatic, and magnetic systems; and springs [20]. The EN 957-5:2009 concerns safety requirements for stationary exercise bicycles and upper-body crank training equipment in addition to the general safety requirements of EN 957-1 [21]. These European standards are similar to the Korean safety criteria annexes.

The ASTM F3021-17 (“Universal Design of Fitness Equipment for Inclusive Use by Persons with Functional Limitations and Impairments”) contains additional requirements not set forth in ASTM standards for the design of commercial fitness equipment to increase access and user independence by people with functional limitations or impairments [12]. It covers indoor fitness equipment in a commercial environment for individuals aged 13 years and above and is aimed at ensuring that the fitness product remains functional and safe when the equipment is operated according to the manufacturer's instructions. This guide includes the scope, reference documents, terminology, color value contrast, design and construction requirements (general requirement and control panels/consoles), keywords, and an annex. However, this is not the standard for specific exercise equipment. Instead, it describes the exercise equipment that is excluded from each category and contains numerical information and figures as guidelines for fitness equipment manufacturers to design their products. Based on these results, we decided to include additional requirements that are not covered by the Korean safety criteria, such as ASTM F3021-17.

### 2.3. Spinal Cord Injury Participants

The number of subjects for this study was selected based on the results of previous studies [22–24], which reported that 80 to 90% of problems related to usability evaluation can be found between 5 and 8 people. In general, severe errors affecting the functionality can be detected in the early stage by recruiting smaller samples. As the process continues, usability issues are harder to find, requiring larger samples. Five subjects with spinal cord injuries were recruited through a recruitment announcement at the National Rehabilitation Center (Table 1). The inclusion criteria were as follows: (1) people diagnosed as having complete injuries of motor function of the types of wither ASIA A or B defined by the American Spinal Injury Association (ASIA) without cerebral injuries and complications such as bone fracture or bedsores, and the injury level of spinal cord injury patients was collected through medical records based on the injury level diagnosed by medical staff based on ASIA's ISCSCI, an international standard; (2) people with spinal cord injury who had normal (5 score) grade or higher of upper-body muscle strength; (3) those between the ages of 20 and 65; (4) those who understood the purpose of usability evaluation and voluntarily agreed to participate; (5) those without orthopedic problems in the shoulder and upper limbs; (6) those who can move around the community on their own; (7) those with an onset of the disability period of longer than 1 year. Pregnant women were excluded. As a result, there were a total of 5 participants, all with thoracic level (T3~T12) spinal cord injury, consisting of 3 males and 1 female using manual wheelchairs, and 1 male using a power wheelchair.

### 2.4. Usability Test of Existing Exercise Equipment

A usability evaluation (IRB No: NRC-2021-04-037) was conducted to identify aspects in need of improvement in existing upper-body exercise equipment [25–28].

#### 2.4.1. Exercise Equipment

**Figure 1:**
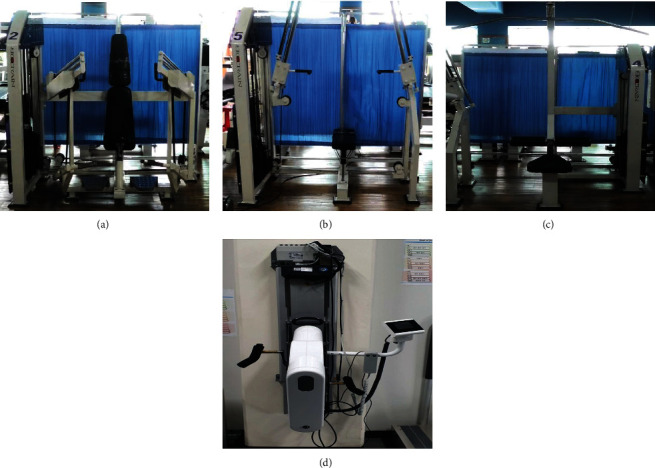
Exercise equipment used for usability evaluation. Strength exercise equipment ((a) seated chest press machine, (b) seated shoulder press machine, and (c) seated lat pull-down machine) and aerobic exercise equipment ((d) arm ergometer).

Especially for individuals with paraplegia with spinal cord injuries, upper-body exercise is necessary to maintain mobility and daily living activities. Regarding the exercise equipment to be evaluated for usability, three types of muscle strength exercise equipment (upper-body exercise: chest, shoulder, and back) focusing on strength and muscular endurance and one type of aerobic exercise equipment (arm ergometer) focusing on developing cardiorespiratory endurance, which are not classified as medical devices, were selected (Figure 1).

#### 2.4.2. Usability Test

The usability evaluation procedure was the following: (1) We explained the purpose of the usability evaluation procedures, precautions when participating, and how to use exercise equipment to the participants. In the preliminary questionnaire, the demographic and disability characteristics of the subjects were investigated. For users who had never used exercise equipment, we demonstrated how to transfer from a wheelchair and how to use the equipment. (2) An “Access-Exercise-Operation-Exit” scenario was performed on four exercise equipment. We measured task performance score (effectiveness) and time (efficiency) based on the observation record sheet (Table 2). The time required to complete the task (regardless of whether it helps or not) was measured once the instructions were completed. According to the degree of task performance, 100 points were given for complete success, 75 and 50 points for partial success, and 0 points for failure. We also recorded the participants' additional opinions. When the subjects asked for help, we provided as much help as they needed, and they were allowed to take a sufficient rest after completing the tasks of each exercise equipment. (3) The questionnaire (Table 3), which evaluated safety and satisfaction, was based on a Likert scale (1-5 points) [29–32].

### 2.5. Data Analysis

The SPSS 24.0 (SPSS Inc., Chicago, IL, USA) for Windows was used for the statistical analyses. The performance scores for four exercise equipment were presented as mean and standard deviation, and safety and satisfaction for four exercise equipment were analyzed using the Kruskal-Wallis test. The significance level was set at *α* = 0.05.

## 3. Results

### 3.1. Usability Test of Existing Equipment

#### 3.1.1. Performance Time and Score Were Measured for Four Types of Upper-Body Exercise Equipment

The results for the 12 tasks are presented in Tables 4 and 5. The “transfer” among tasks took the most time for all three types of muscle strength exercise equipment, and we observed a large variation depending on individual characteristics (degree of damage and usual exercise experience). In addition, for the seated shoulder press machine and seated lat pull-down machine without back support, there were many cases requiring help for holding the handle or exercising. In the case of the arm ergometer, users could exercise while using it in a wheelchair; therefore, they could use it without difficulty, and the variation according to individual characteristics was relatively small.

#### 3.1.2. The Safety Results for Four Types of Upper-Body Exercise Equipment

Table 6 shows that the seated chest press machine had the lowest score in terms of safety, while the arm ergometer scored the highest. In particular, in the case of a chest press machine, the transfer and contact safety scores are low owing to its structure. As a result of obtaining the average value for each item for four types of upper-body exercise equipment, values smaller than 3.0 were also found for the transfer and contact safety. However, it was not statistically significant (*p* > 0.05).

#### 3.1.3. The Satisfaction Results for Four Types of Upper-Body Exercise Equipment

**Figure 2:**
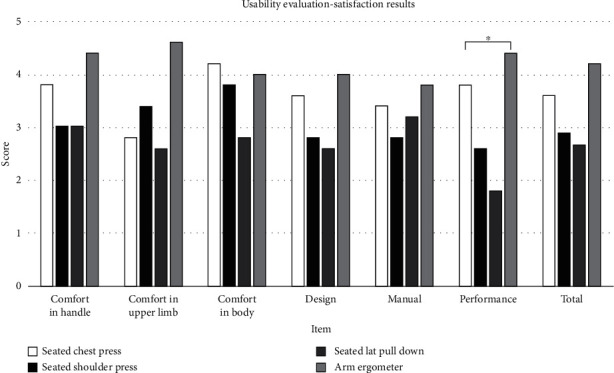
Usability evaluation-satisfaction results.

In terms of satisfaction (Table 7 and Figure 2), the seated lat pull-down received the lowest score. Overall, the Likert score on whether the body posture was well maintained and felt comfortable during exercise was low; as a result, satisfaction with exercise performance was the lowest (*p* < 0.05).

#### 3.1.4. Identifying Areas for Improvement through Evaluations

**Figure 3:**
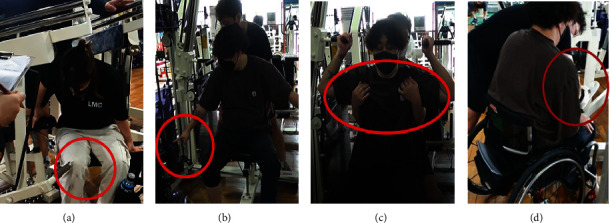
Main accessibility aspects in need of improvement. (a) There is a risk of colliding with frame or other structural parts of the equipment when transferring to get in and out of the equipment. (b) It is difficult to adjust the weight owing to the distance between the weight stack units and the seat. (c) It is difficult to maintain the safety of the upper body during exercise execution. (d) It is difficult for users to lift the lower body by themselves and pass the leg to the opposite side.

From the usability evaluation and the subjects' opinions, we derived the following aspects in need of improvement (Table 8 and Figure 3).

#### 3.1.5. Multidisciplinary Team Meeting

The first advisory meeting addressed the direction of the guidelines. The main comments were as follows: (1) the definition of universal needs clarification; (2) the range of exercise equipment to be included in the guidelines must be selected; (3) the guidelines should be decided into essential and recommended elements; (4) it is necessary to fully review whether the manufacturer of the exercise equipment is acceptable.

The second advisory meeting regarded the development of universal exercise equipment suitable for the situation in Korea. The main opinions were as follows: (1) it is recommended to include content on the development of exercise equipment that applies universal principles (equitable use, flexibility in use, simple and intuitive use, perceptible information, tolerance for error, low physical effort, size, and space for approach and use); (2) guidelines considering both outdoor and indoor exercise equipment are needed.

The main opinions of the third advisory meeting were as follows: (1) it is better to leave it as a recommendation for items that require numerical representation, as universal standards can be difficult to tailor in detail for every product or facility; (2) it is necessary to first present the basic principles and then add the details.

The contents of the fourth advisory meeting were as follows: (1) since the usability evaluation of upper-body exercise equipment was performed, it is necessary to include this first before expanding the range of exercise equipment in the future; (2) the purpose of guideline is not to manufacture customized equipment for people with disabilities; (3) for the purpose of physical education for daily recovery of people with disabilities, it is necessary to include various exercise equipment types so that they can exercise not only large muscles but also light inner muscles; (4) it is necessary to produce figures based on the Korean human body standards.

## 4. Discussions

The subjects who participated in this study were 5 patients with complete paralysis due to pleural effusion injury. In particular, the physical abilities of pleural numbers 2 to 12 include normal motor function in the head, neck, shoulders, arms, hands, and fingers, and the ability to control the rib muscles, chest muscles, or torso. At T10 to T12 levels, trunk control is further improved due to increased abdominal strength [33]. The injury level of spinal cord injury patients was collected through medical records based on the injury level diagnosed by medical staff based on ASIA's ISCSCI, an international standard. The ASIA test determines motor level by examining 10 major muscles on each side of the body using manual muscle testing (MMT), which is graded from grade 0 (complete paralysis) to grade 5 (normal), and sensory level is determined by sensory examination of each of the 29 dermatomes of the major sensory points on each side of the body. Sensory tests are pin-prick and light touch and are graded on a scale of 0 (no sensation) to 2 (normal). Sensory tests include perianal tests and sphincter tightening tests, and the diagnostic results are classified into bilateral sensory and motor levels and neurological levels. This study was based on the level of neurological damage, which refers to bilateral damage to the lowest segment of the spinal cord. It refers to a state in which normal sensation and antigravity motor function are preserved [34].

Accordingly, in this study, five subjects who met the selection and exclusion criteria were targeted among those who applied after seeing the recruitment notice for T2-T12 level paraplegia and pleural effusion injury who had no problems with hand function and had difficulty with trunk control. In addition, since exercise is an important factor for those with spinal cord injury, regardless of age or gender, factors based on gender were not considered, and the study was conducted on people with spinal cord injury who use wheelchairs. Wheelchairs included both power and manual wheelchairs, and this was because subjects were selected who had mobility to the exercise program site in order to move around the community on their own and maintain their health. Anthropometric measurements in this study were conducted before usability evaluation, height was measured on a tilt table, and weight was measured by subtracting the wheelchair from the total weight.

For disabled people who have less daily activities than nondisabled people, regular exercise is essential to maintain physical health and prevent secondary complications caused by disability. However, as a result of the 2020 survey on the disabled, 60.7% of all disabled people were overweight, an increase of 6.4% compared to 2017 [35]. And according to the 2020 report on the participation rate in daily sports by the Ministry of Culture, Sports and Tourism, the proportion of disabled people exercising more than twice a week (more than 30 minutes per time) was 24.3%, a decrease of 0.7% compared to 2019. Lack of facilities and difficulty in moving were cited as reasons why disabled people cannot exercise [36]. Among the disabled, it was confirmed in the interview that people with spinal cord injuries are reluctant to visit the gym because they use wheelchairs and have difficulty accessing exercise equipment and reception desks while in a wheelchair.

In this study, through usability evaluation and consultation with stakeholders, we derived points to consider in the guidelines for accessible exercise equipment.

It is necessary to consider how wheelchair users access the exercise equipment. The safety results for existing equipment showed a low score (<3.0) for the transfer and contact. If there is no exercise equipment that can be used while riding in a wheelchair, consideration should be given to enable access from various directions according to the user's characteristics, namely, the position where transfer is easier. If possible, the risk of bumping into the equipment when approaching should be eliminated by ensuring that there are no parts protruding from the exercise equipment or surrounding floor. Currently, in the case of domestic gymnasiums, barrier-free criteria are not applied, and there are no regulations on the placement of exercise equipment. Therefore, the access method to the exercise area (facility regulations, auxiliary equipment to be provided, etc.) and placement of exercise equipment should also be considered.

Information such as explanations of the exercise equipment and how to use it should be located within the wheelchair user's field of vision. In most of the existing exercise equipment, the instructions engraved on it were adjusted to the eye level of the standing people without disabilities. To accurately use the exercise equipment in the direction intended by the manufacturer and increase the effect of exercise, it is important to consider the visual field of the user, because information confirmation must precede exercise. Only one out of five participants in the usability evaluation conducted in this study knew how to use the exercise equipment. Considering the participation rate in sports for people with disabilities in Korea, it is necessary to explain the exact exercise equipment and exercise method.

It is also necessary to consider how wheelchair users transfer from the wheelchair to the seat of the exercise equipment. It should be possible to adjust the height so that there is no large difference in seat height between the wheelchair and the exercise equipment; if the space necessary for transfer is secured and auxiliary handles are provided, it will be possible to transfer more safely. Among the usability evaluation results, transfer took the most time for the three strength exercise equipments, and external assistance was needed. Therefore, this should be considered in the guidelines so that transfer can be performed alone. In addition, since professional support is rarely assigned to gymnasiums in local communities in Korea, it would be better if adapted sports experts present videos that show how to transfer safely, for example, using in QR codes.

Parts that require manipulation (levers, handles, buttons, and Velcro) of each exercise equipment must be within the wheelchair user's range of motion. Currently, in the case of commercial exercise equipment, there are regulations on the range of available physical information for people without disabilities, but it will be necessary to conduct experiments to prepare the adequate conditions for people with disabilities who have difficulty balancing their upper body alone.

In the case of commercial exercise equipment in Korea, even though it was developed for people without disabilities, it is possible to use it with a structure that supports the body during exercise alone. However, when there was no support, the user also complained of anxiety during the exercise, and there was a possibility of a safety accident. Therefore, various supports or assistive devices that provide body support according to each piece of equipment are needed. To this end, the experiences of various stakeholders, such as rehabilitation sports experts, clinical experts, and exercise equipment experts, are important.

Exercise equipment that can be used while riding a wheelchair must be developed considering standard wheelchair specifications. In addition to the wheelchair's own brake, it is necessary to provide a fixing device so that the wheelchair does not move during the exercise.

According to the Disability Discrimination Act, all people with disabilities have equal rights. Therefore, the purpose of this study is to improve the accessibility of exercise equipment so that people with disabilities can more easily manage their health at a gym of their choosing, similar to people without disabilities. However, it is impossible for rehabilitation exercise professionals to exist in all gyms of the community. Therefore, it would be useful to create a guide that allows people with different types of disabilities to safely exercise.

When designing exercise equipment, it is necessary to understand how to use it more intuitively. By referring to universal principles, exercise equipment can be designed so that both people with and without disabilities can use it well.

Exercise is important and provides many benefits to people with and without disabilities. An increase in activity levels decreases secondary conditions and health costs, often associated with disability. Providing people with disabilities, the opportunity to exercise in public facilities, rather than in specialized medical therapy settings, increases the social opportunity to interact with family members and friends. Therefore, it is necessary to develop universal guidelines, which can be directed by the results of our usability evaluation. The results of this study are expected to help people with disabilities maintain their health and improve their quality of life by enabling them to access the exercise equipment more easily.

However, there are some limitations in this study. (1) The universal design policies show different development patterns according to sociocultural or political characteristics of each country. Therefore, it is necessary to investigate universal exercise equipment standards in more diverse countries in future works. Based on various literature reviews, it will be possible to draw more general requirements for improving the accessibility of exercise equipment for the disabled as well as the characteristics of each country. (2) The design of the usability evaluation was mainly based on observation (the performance score and time) and a questionnaire survey. In the future, it is necessary to add other objective detection methods, such as the detection of EMG and IMU signals that can provide more objective judgments. (3) The usability evaluation did not include diverse equipment and disability types. It is needed to expand the range of accessible exercise equipment (fixed-type training equipment, bench presses, treadmill, bicycle ergometer, stepper, rowing machines, exercise, and so on) for different disability types in the future. Because spinal cord injury has diverse functions depending on the level of injury, future research should include more subjects depending on each level of spinal cord injury and gender and conduct usability evaluation to generalize the results. It is necessary to derive numerical values through a thorough usability evaluation based on the Korean body shape standard. Korean exercise equipment developers are yet to actively consider people with disabilities in equipment development. Therefore, the universal guidelines are a recommendation rather than a regulation, and it will be necessary to consider people with disabilities in the early stages of design and development. Moreover, it is believed that a system that certifies well-performing companies, such as IFI, will be beneficial.

## 5. Conclusions

In this study, we aim to improve the accessibility of exercise equipment for the disabled. Firstly, we conducted the standard review related to exercise equipment. Then, the usability evaluation was performed on 4 types of existing upper-body exercise equipment for individuals with spinal cord injuries. In the usability evaluation, task performance score and time were measured, and questionnaires were conducted. Based on the results, we intend to derive the points in need of improvement. From the convergence of literature, results of the usability evaluation, and opinions of various stakeholders, we proposed the 8 requirements in universal viewpoints. In the future, it is necessary to perform usability evaluation by expanding the type of disability and the type of exercise equipment. In addition, the guideline direction was derived, but considering the people who design exercise equipment, it will be necessary to conduct a usability evaluation targeting more users to obtain numerical information suitable for domestic users. Through the results of this study, it is expected that it will help people with disabilities to maintain their health and improve their quality of life by enabling them to access exercise equipment more easily.

## Figures and Tables

**Table 1:** General characteristics of individuals with thoracic SCI.

Subject	Sex	Age	Height (cm)	Weight (kg)	Level of injury	AIS scale	Type of wheelchair	SCIM-III
1	F	28	163	55	T3	B	Manual	57
2	M	28	180	100	T5	A	Manual	46
3	M	50	170	80	T9	A	Power	38
4	M	46	172	60	T10	A	Manual	48
5	M	44	168	80	T12	A	Manual	50

**Table 2:** “Access-Exercise-Operation-Exit” scenario record sheet.

Task	Function for the task	
Access	Approach/wheelchair securement	After approaching the equipment, secure the wheelchair
	Information confirmation	Check the information on the characteristics of the equipment, exercise method, etc.

Exercise	Transfer	Transfer from the wheelchair to the correct start position for the exercise equipment
	Fix the body	Fix the body on the equipment and grip the handle
	Preparatory exercise	Perform 1-3 exercise movements
	Perform exercise	Perform 10 exercise movements

Operation	Height adjustment	(Optional) Adjust the height of equipment in 2 steps
	Change weight	Adjust the amount of weight in 2 steps
	Check feedback	(Optional) Check the feedback (exercise results)

Egress	Release the fixture	Release the fixture
	Transfer	Transfer from the equipment to the wheelchair
	Egress	Get out of the equipment

**Table 3:** Safety and satisfaction questionnaire.

	Category	Details
Safety	Safety in use	There is no risk of falling during the exercise
	Safety in transfer	There is no risk of falling when getting on/off the equipment
	Safety in contact	There is no risk of bumping into the surrounding structures or injuring parts of the body when using the equipment
	Safety in speed	There is no risk due to the initial speed
	Safety in weight	There is no risk due to the initial weight

Satisfaction	Comfort in handle	It is comfortable to hold the handle
	Comfort in upper limb	The range of motion during exercise is appropriate, so the shoulders and arms are comfortable
	Comfort in body	The range of motion during exercise is appropriate, so the body is comfortable
	Design	Satisfied with product color and design
	Manual	Equipment instruction manual is easy to understand
	Performance	The range of motion, weight, and speed during exercise are appropriate, and the exercise is well-performed

**Table 4:** Result of the performance time.

Task		Performance time (sec) (mean (SD))			
		a	b	c	d
Access	Approach/wheelchair securement	13.8 (3.82)	10.0 (6.23)	13.8 (8.08)	9.2 (2.04)
	Information confirmation	25.6 (6.74)	23.6 (9.26)	22.8 (6.88)	7.6 (3.83)

Exercise	Transfer	67.8 (54.36)^∗^	49.6 (27.13)^∗^	44.2 (51.81)^∗^	—
	Fix the body	4.6 (1.85)	1.6 (0.49)	11.0 (2.61)	2.4 (0.80)
	Preparatory exercise	11.8 (6.37)	16.0 (9.32)	14.2 (5.19)	7.8 (1.47)
	Perform exercise	28.4 (10.38)	36.25 (16.0)	37.4 (9.39)	15.6 (3.26)

Operation	Height adjustment	—	—	—	7.8 (6.31)
	Change weight	11.4 (7.58)	19.0 (11.02)	6.2 (1.60)	—
	Check feedback	—	—	—	10.4 (5.00)

Egress	Release the fixture	—	—	—	10.4 (5.00)
	Transfer	46.6 (36.36)^∗^	34.6 (21.10)	43.2 (33.91)^∗^	—
	Egress	9.6 (2.73)	4.4 (1.96)	4.4 (2.06)	3.6 (0.80)

*N* = 5; ^∗^mean time > 40 sec. SD: standard deviation; a: seated chest press machine; b: seated shoulder press machine; c: seated lat pull-down machine; d: arm ergometer.

**Table 5:** Result of the performance score.

Task		Performance score (mean (SD))			
		a	b	c	d
Access	Approach/wheelchair securement	80.0 (10.00)	100 (0.00)	95.0 (10.00)	100 (0.00)
	Information confirmation	100 (0.00)	100 (0.00)	100 (0.00)	100 (0.00)

Exercise	Transfer	65.0 (20.00)^∗^	80.0 (24.49)	85.0 (12.25)	—
	Fix the body	95 (10.00)	100 (0.00)	60.0 (20.00)^∗^	100 (0.00)
	Preparatory exercise	85 (20.00)	85 (20.00)	75.0 (22.36)^∗^	100 (0.00)
	Perform exercise	85 (20.00)	75.0 (38.73)	75.0 (22.36)^∗^	100 (0.00)

Operation	Height adjustment	—	—	—	100 (0.00)
	Change weight	100 (0.00)	80 (40.00)	100 (0.00)	—
	Check feedback	—	—	—	80.0 (10.00)

Egress	Release the fixture	—	—	—	80.0 (10.00)
	Transfer	70.0 (18.71)^∗^	80.0 (24.49)	75.0 (22.36)^∗^	—
	Egress	100 (0.00)	100 (0.00)	100 (0.00)	100 (0.00)

*N* = 5; ^∗^partial success score < 75 score. SD: standard deviation; a: seated chest press machine; b: seated shoulder press machine; c: seated lat pull-down machine; d: arm ergometer.

**Table 6:** The safety results for four types of upper-body exercise equipment.

	Total	Safety (mean (SD))				
		a	b	c	d	*p*
Total	3.14 (0.95)	2.72 (0.81)	2.96 (1.18)	3.20 (1.16)	3.68 (0.48)	0.376
Safety in use	3.40 (1.64)	3.40 (1.14)	3.20 (2.05)	3.00 (1.87)	4.00 (1.73)	0.816
Safety in transfer	2.90 (1.74)	1.60 (1.34)	2.60 (1.52)	3.20 (1.64)	4.20 (1.79)	0.070
Safety in contact	2.85 (1.50)	2.40 (1.67)	3.20 (0.84)	3.20 (1.30)	2.60 (2.19)	0.800
Safety in speed	3.00 (1.41)	2.80 (0.83)	3.00 (1.22)	2.80 (1.92)	3.40 (1.82)	0.933
Safety in weight	3.55 (1.28)	3.40 (1.34)	2.80 (1.64)	3.80 (1.10)	4.20 (0.84)	0.454
*p*	0.416	—	—	—	—	—

*N* = 5. SD: standard deviation; a: seated chest press machine; b: seated shoulder press machine; c: seated lat pull-down machine; d: arm ergometer.

**Table 7:** The satisfaction results for four types of upper-body exercise equipment.

	Total	Safety (mean (SD))				
		a	b	c	d	*p*
Total	3.34 (1.13)	3.60 (0.82)	2.90 (1.37)	2.67 (1.22)	4.20 (0.46)	0.105
Comfort in handle	3.55 (1.28)	3.80 (0.84)	3.00 (1.58)	3.00 (1.58)	4.40 (0.55)	0.279
Comfort in upper limb	3.35 (1.46)	2.80 (1.79)	3.40 (1.14)	2.60 (1.52)	4.60 (0.55)	0.103
Comfort in body	3.45 (1.32)	4.20 (0.84)	3.80 (1.30)	2.80 (1.64)	4.00 (0.71)	0.228
Design	3.25 (1.25)	3.60 (0.89)	2.80 (1.30)	2.60 (1.52)	4.00 (1.00)	0.256
Manual	3.30 (1.17)	3.40 (0.89)	2.80 (1.48)	3.20 (1.10)	3.80 (1.30)	0.609
Performance	3.15 (1.53)	3.80 (1.30)	2.60 (1.82)	1.80 (0.84)	4.40 (0.55)	0.040^∗^
*p*	0.917	—	—	—	—	—

*N* = 5; ^∗^significant at *p* < 0.05. SD: standard deviation; a: seated chest press machine; b: seated shoulder press machine; c: seated lat pull-down machine; d: arm ergometer.

**Table 8:** Identifying area for improvement through evaluations.

Task	Problem revealed through usability evaluation
Approach/egress	There is a risk of colliding with frame or other structural parts of the equipment when transferring to get in and out of the equipment.
Information confirmation	The text on the exercise information is difficult to see, and the explanation of how to use the equipment is insufficient.
Approach	A movable chair is required.
Approach	The space is insufficient for wheelchair access to the exercise equipment.
Transfer	It is difficult for users to lift the lower body by themselves and pass the leg to the opposite side.
Transfer	The transfer is difficult because of the height difference between the wheelchair and the equipment seat.
Exercise execution	It is difficult to maintain the safety of the upper body during exercise execution.
Wheelchair securement during exercise	When using a wheelchair during exercise, the user needs a device to secure the wheelchair to the floor.
Change weight	It is difficult to adjust the weight owing to the distance between the weight stack units and the seat.
Release the fixture	It is difficult to remove or fasten parts of the equipment.

## Data Availability

The demographics and clinical data collected to support the findings of this study are restricted by the Ethics Committee of the Province of National Rehabilitation Center (Republic of Korea) in order to protect patient privacy. Data are available from Kwang-Ok An, National Rehabilitation Center 58, Samgaksan-ro, Gangbuk-gu, Seoul, Republic of Korea (anko04@korea.kr), for researchers who meet the criteria for access to confidential data.
